# Testicular development induced by GnRH-IS in budgerigar (*Melopsittacus undulatus*)

**DOI:** 10.21451/1984-3143-AR2019-0103

**Published:** 2020-02-11

**Authors:** José Antonio Herrera-Barragán, Samantha Anahí Carcoba-Pérez, Juan José Pérez-Rivero, Alejandro Ávalos-Rodríguez, Ana Karen Vargas-Ibarra, Fernando Gual-Sill, Osvaldo López-Díaz

**Affiliations:** 1 Departamento de Producción Agrícola y Animal, Universidad Autónoma Metropolitana Unidad Xochimilco, México; 2 Programa de Maestría en Ciencias Agropecuarias, Universidad Autónoma Metropolitana Unidad Xochimilco, México

**Keywords:** ultrasonography, parakeet, male reproduction

## Abstract

Nowadays, the third part of parrots in the world is endangered or vulnerable; an alternative for their preservation is assisted reproduction in captivity through hormonal manipulation. In birds, GnRH is the main hormone which controls reproductive physiology, it is known there are three types: GnRH-I, GnRH-II and GnRH-III, involved in the release or inhibition of luteinizing hormone and follicle stimulant hormone to control gonadal and gametic development. The objective of this study was, to evaluate the effect of administrating synthetic GnRH-I in the testicular development of *Melopsittacus undulatus*. Twenty-eight adult budgerigars were randomly divided in two groups: control (n=14) and treated (n=14) with a unique dose of synthetic GnRH-I. Testicular development was assessed through ultrasonography and density was evaluated with pixels. Germinal diameter and thickness of germinal epithelium were determined with histology; there were identified and countified different cellular strains in seminiferous tubules therefore spermatobioscopy. Results. Ecographic density was: control group: 76 ± 7 pixels, treated group 41 ± 3 pixels. Thickness of germinal epitellium, 51.5 ± 2.9µm and 73.1 ± 3.1µm, for control group and treated group respectively. Sperm concentration in the treated group was 300% superior than in control group. It is concluded that the administration of synthetic GnRH-I, is a viable alternative to be used as part of the assisted reproductive techniques to induce reproduction.

## Introduction

Avian species are very diverse, as well as their life patterns and reproductive strategies, due to this diversity, it is difficult to determine the environmental factors that regulate their reproductive activity. They exist in captivity around 500 avian species (SEMARNAT, 2010). However, the reproduction of these birds is irregular, associated with an inadequate activation of the hypothalamus-hypophysical-gonada axis (Lugo, 2011).

Birds have different seasonal reproductive mechanisms, one of these is associated to photoperiods cycles, due to fact that they perceive light through photo receptors which are in the retina and pineal gland (Nakane and Yoshimura, 2019). Activation of photo receptors induce the secretion of thyroid stimulant hormone (TSH) which activates thyroid hormone in the hypothalamus controlling the secretion of gonadotropin release hormone (GnRH) and as a result, the secretion of luteinizing hormone (LH) and follicle stimulant hormone (FSH), which stimulate gonadal growth, as well as gametes productions (Bentley et al., 2006; Nakane and Yoshimura, 2019).

Studies developed in birds have demonstrated the presence of GnRH release factors; GnRH-I, which has a molecular structure similar to mammals’ GnRH, which has a more long-lasting effect on gonadotropins (LH and FSH), while GnRH-II is released in the birds’ non reproductive season, being 2.5 times more powerful than GnRH-I, acting on LH producing negative feedback to GnRH-I (Peralta and Miazzo, 2002). There are also studies where it has been reported that GnRH-III exists, even it has not been completely characterized (Peralta and Miazzo, 2002).

There have been developed synthetic analogs and antagonists of GnRH which have a highly specific activity in different reproductive tissues (Ottinger et al., 2002). There are synthetic GnRH-I analogs (GnRH-IS) commercially available, which have a biological effect when linking with the receptor of GnRH in the adenohypophysis (Paredes, 2010).

Reproduction of parakeets is important worldwide from the point of view of preservation and sustainable economic use (Lugo, 2011). Nevertheless, there have not been developed protocols of assisted reproduction in a specific species matter, knowing also that parakeets reproduction is particularly difficult due to its susceptibility to stress (Bradshaw and Engebretson, 2013).

On the other hand, considering the economic impact that the pets business has, which can be achieved through reproduction in captivity, it has been used as a model to be development and implementation the assisted reproduction techniques to specimens of *M. undulatus,* which could be extrapolated to other endangered parakeet species, as their reproductive characteristics are similar in order to favor parakeet production, because since the 70s 7.5 million of wild birds were exported leaving an economic spill of 500 U.S. dollars per bird (Costantini et al., 2009; Calderón et al., 2015 ; Whelan et al., 2015).

For the above mention, the objective of this study was to evaluate the effect of the administration of exogen GnRH-IS in gonadal development and spermatozoa production in *M. undulatus.*

## Methods

The study was developed during autumn (august-October) in laboratories of the Universidad Autonoma Metropolitana Campus Xochimilco, in the central region of Mexico (19°18’08.3”N 99°06’11.2”W). Twenty-eight male adults of *M. undulatus* (32.0 ± 2.7g body weight) were used, clinically healthy, with 1 to 2 years old. Specimens were kept individually in bird cages sized 45 cm x 30 cm x 30 cm. They were fed with a mixture of seeds (*Panicum miliaceum* 40%, *Phalaris canariensis* 10%, *Avena sativa* 10%) and potable water *ad libitum,* through the whole experiment, they were kept in a photo period of 12 light hours and 12 dark hours. Before the beginning of the study, all birds had a 30 days period of adaptation to the same conditions. Requirements established in Norma Oficial Mexicana NOM-062-ZOO-1999 of Technical specifications for production, care and use of laboratory animals were accomplished.

They were randomly integrated 14 specimens to each group: control group (CG) and treated group (TG). Each one of the budgerigars of TG received a unique dose of 3µg of GnRH-IS (Relina 500™ [Gonadorelina] PiSA Agropecuaria, Mexico), subcutaneous in the fold of the left wing; the determinations of the study were done 5 days post treatment.

### Seminal obtention and evaluation

Ejaculates from specimens of CG and TG were obtained through dorsoventral massage (Herrera et al., 2013). Each ejaculated was deposited in an Eppendorf tube with 20 µl of Lake extender (sodium glutamate 0.1135 M, fructose 0.04 M, potassium acetate 0.050 M and magnesium acetate 0.0056 M) for its evaluation (Herrera et al., 2013). Sperm concentration was estimated with a Neubauer chamber and an optic microscope Optisum™, also the percentage of spermatozoa with movement was determined at a temperature of 37 °C; in a smear prepared with eosin/nigrosine the percentage of alive spermatozoa and its morphology was determined (Herrera et al., 2013).

### Ultrasonography

The specimens of CG and TG were induced to general anesthesia with isoflurane (SofloranVet™ Pisa Agropecuaria, Mexico) at 2.5% in oxygen, they were monitored through the whole process with an oximeter OX 100™ (KONTROLab, Italy). Testicular size and density was determined for every specimen through testicular ultrasonography in mood “M”, using a K10 Ultrasound™ (KONTROLab, Italy) and a transductor of 6.5 MHz positioned in the dorsal area of the bird, on the pelvis region to have an approximation with testicles (Melnychuk et al., 2002). During the study we obtained images, which were analyzed with the ImageJ software (National Institutes of Health, 2019) randomnly chosing 10 points of 2.0 mm^2^ of each test obtaining the values of pixel intensity (Moxon et al., 2015).

### Testicular obtention and evaluation

At the end of the ultrasonography evaluation, 3 birds from each group were randomly chose, which were euthanatized, with an intra thoracic overdose of sodium pentobarbital (100 mg/kg). The testicles were obtained by dissection, which were fixed in Bouin solution and processed in a conventional way in paraffin; 3 semi-serial slides (2,4,6 cut) were obtained at a thickness of 3 µm, these slides were stained with hematoxylin-eosin (Iseki et al., 2018; Prophet 1994).

### Histological evaluation of testicles

Using an optic microscope Optisum™, 10 microscopic fields were observed at 200X and 400X per slide of each bird. With a camera (OP900 Optisum™), images were obtained. Through a free access software LSM5 (Carl Zeiss) it was possible to obtain the area of seminiferous tubules and thickness of germinal epithelium. Also, there were morphological identified and quantified the spermatogonium, spermatocytes, round spermatids, elongated spermatids, Sertoli cells and Leydig cells (Hänse et al., 2008; Perez-Rivero et al., 2014).

### Statistical analysis

Normality of variables was verified with a Shapiro-Wilks test; it was done descriptive statistics, with the Mann-Whitney test the medians of all parameters between CG and TG were compared, taking into consideration p<0.05 as the significance value. Finally, a Spearman lineal correlation was done to compare between groups the density in pixels of germinal epithelium with the sperm concentration per ejaculated. All tests were done in the PAST 3.18 software (Hammer et al., 2001).

## Results

### Basic spermatic evaluation

The sperm concentration in ejaculates of birds of the TG was of 7.7 ± 3.7 X 10^6^ sperm/ml versus specimens of CG, which was of 2.2 ± 0.9 X 10^6^ sperm/ml, making evident a significant difference (p<0.05), the other evaluated parameters did not showed differences, as shown in Table 1.

**Table 1:** Parameters of basic seminal evaluation.

	**CG**	**TG**	**P value (Mann-Whitney)**
% Motility	60 ± 7.4	70 ± 7.3	P>0.05
% Viability	97 ± 1.0	98 ± 1.0	P>0.05
% Abnormal morphology	2.0 ± 1.0	1.0 ± 0.0	P>0.05
Sperm concentration ×10^6^ sperm/ml	2.2 ± 0.9	7.7 ± 3.7	P<0.05

Total medians of basic seminal evaluation (median ± S.D.) per group, Mann Whitney (P*<*0.05).

### Testicular dimention and density by pixels

The testicular area obtained by ultrasonography, in specimens of CG for right testicle was of 22.6 ± 5.5mm^2^ and for left testicle 25.3 ± 6.7 mm^2^ while in TG, the area for right testicle was of 47.6 ± 8 mm^2^ and for left testicle 46 ± 7.9 mm^2^ (p<0.05).

Results obtained of intensity in pixels (Pix) were, for TG of 41.3±3 Pix, and for CG of 76±7 Pix (P<0.001). Generally in testicles of birds of TG, it was observed that they showed regions of heterogeneous testicular parenchyma, with prominent anechoic stippling (Figures11B).

**Figure 1:**
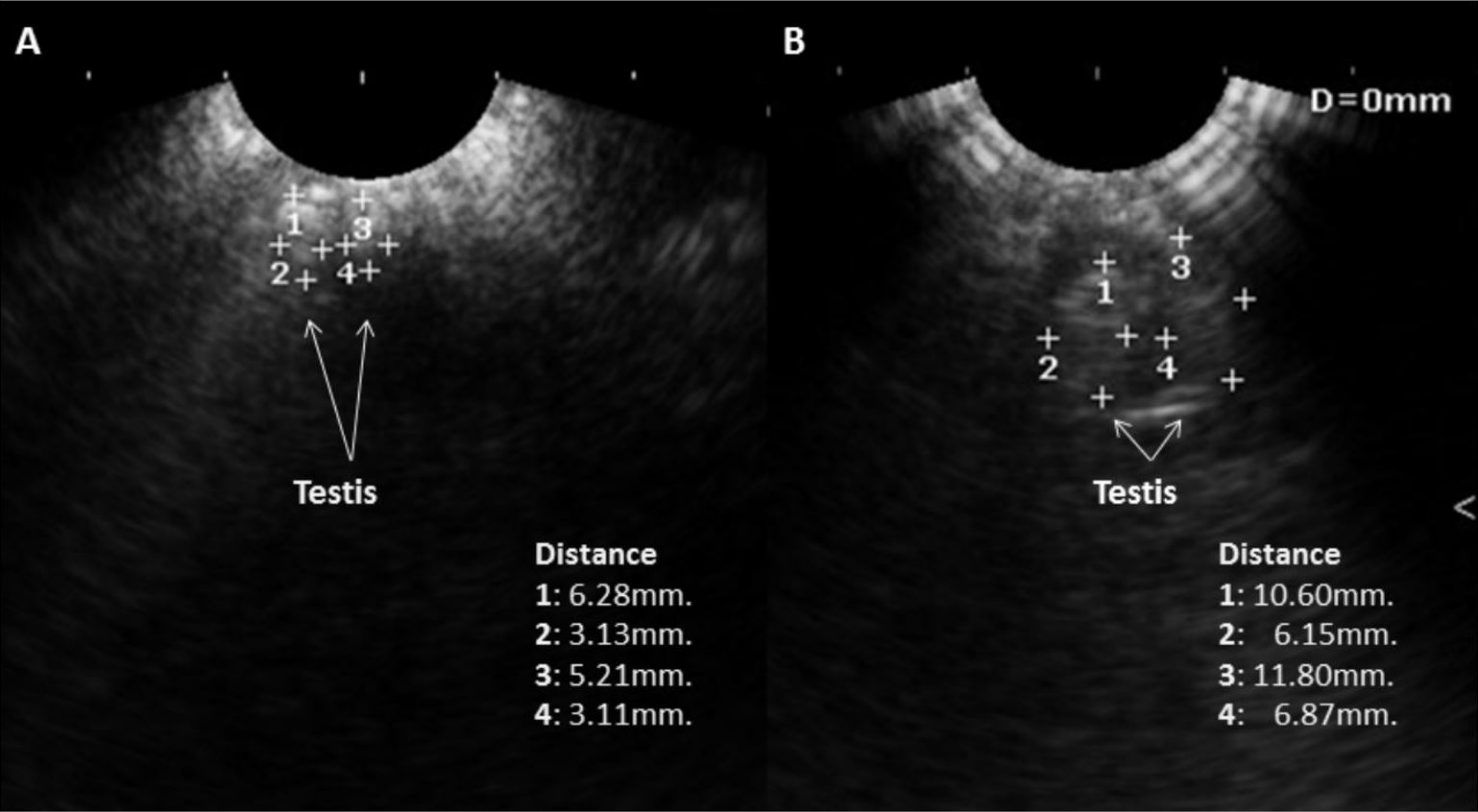
Ultrasonographic image of the testicles of the control group (A) and treated group (B). The (+) associated with 1 and 3 indicate the cranial position of each testicule and the distance to the opposite (+) indicates the length. The (+) associated with 2 and 4 and the distance to the opposite (+) indicates each testicule width.

### Lineal correlation between testicular density and sperm concentration

The evaluation of ultrasonographic images based on pixels, was correlated with the sperm concentration, obtaining as a result that the lower pixel intensity is associated with greater sperm production R=0.816 (p<0.05).

### Histological evaluation of testicles

**Figure 2:**
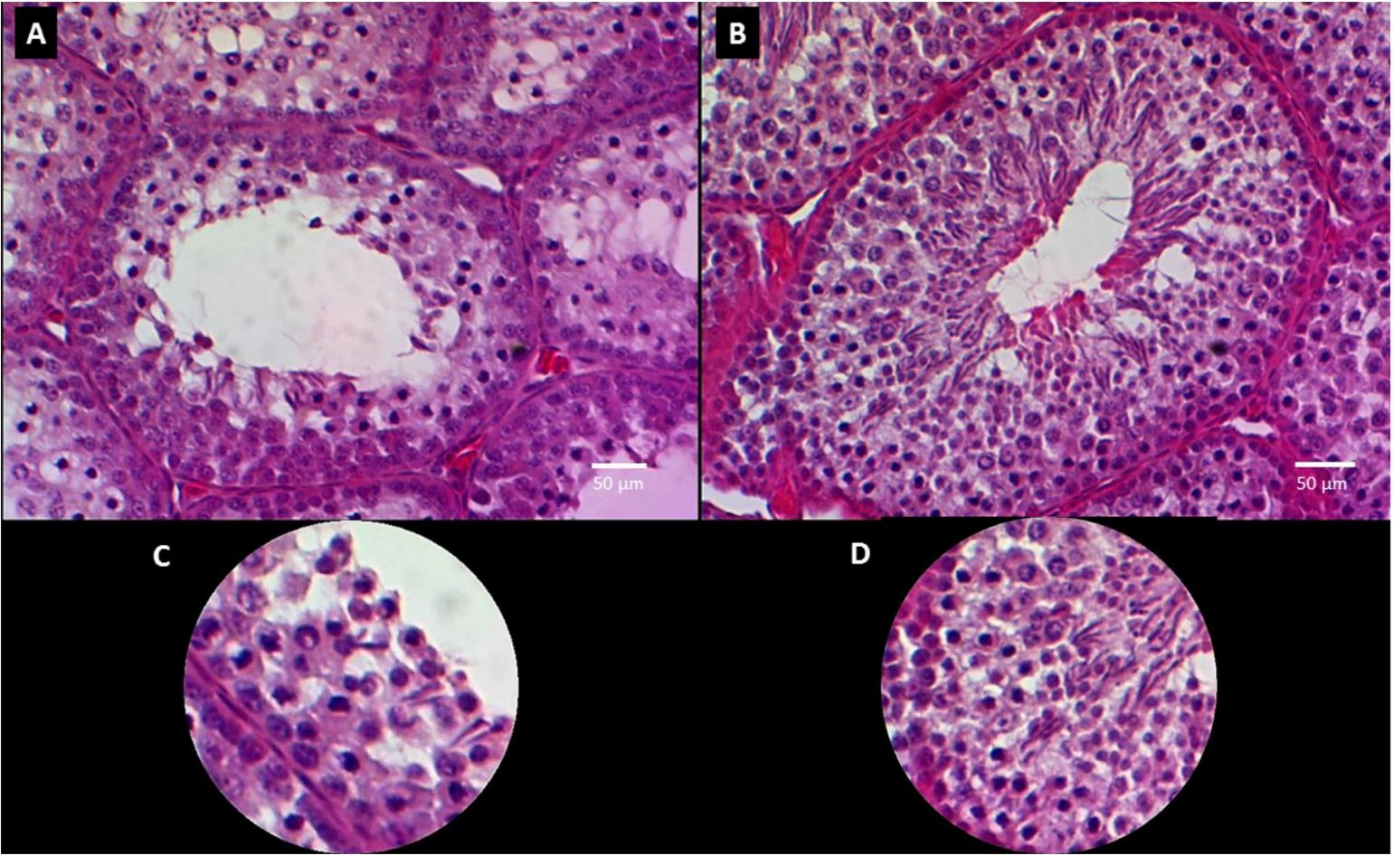
Histologic slides of test at 400X. A: the seminiferous tubule in birds of the control group show lower development of the germinal epithelium B: the seminiferous tubule of birds of the treated group show greater development of germinal epithelium. C: close up of the seminiferous tubule of birds of the control group D: close up of the seminiferous tubule of birds of the treated group.

The measurements obtained of thickness of germinal epithelium and number of germinal cells showed a significant increase in animals of the TG compared with specimens of CG (p<0.05), as shown in Figure 2 and Table 2.

**Table 2:** Morphometric parameters of histological morphology of testicles.

**Number of cells**	**CG**	**TG**	**P value (Mann-Whitney)**
Area of left test (mm^3^)	25.3 ± 6.7	46 ± 7.9	p<0.005
Area of right test (mm^3^)	22.6 ± 5.5	47.6 ± 8	p<0.005
Spermatogonium	33 ± 4.0	58 ± 2.0	p<0.001
Spermatocytes	34 ± 2.0	52 ± 2.0	p<0.001
Sertoli cells	4.0 ± 1.0	6.0 ± 1.0	p<0.001
Leydig cells	3.0 ± 1.0	6.0 ± 1.0	p<0.001
Round spermatids	46 ± 7.0	98 ± 6.0	p<0.001
Elongated spermatids	20 ± 2.0	74 ± 5.0	p<0.001
Area of seminiferous tubules (µm^2^)	67.8 ± 9.4 × 10^3^	71.4 ± 3.9 × 10^3^	p>0.05
Thickness of seminiferous tubules (µm)	51.5 ± 2.9	73.1 ± 3.1	p<0.001

Cellular count (median ±S.E.) per group, during the experimental period. Data showing significant difference (P< 0.05) for the Mann Whitney statistical analysis.

## Discussion

The basic spermatic evaluation, showed an increase of the sperm concentration on TG compared with CG, providing evidence that the treatment with GnRH-IS favors the formation of gametes, these results meet the results reported by Calderón et al. (2015) who reported that with the administration of two different analogs of GnRH viability and sperm concentration of treated specimens were improved and with Hänse et al. (2008) who evaluated the spermatic production of *M. undulatus* during the annual cycle, finding that in reproductive season when GnRH is produce naturally, it is an increase in sperm viability of up to 98%, reporting also an increase in sperm concentration.

It has been proved that stimulation with gonadotropins induces cell activity in mammals’ testicles (Ramaswamy et al., 2017), the results obtained in this study meet the above mention, as the administration of GnRH-IS in *M. undulatus*, induced cellular proliferation in the testicular parenchyma.

Additionally, it was possible to associate through a lineal correlation that lowers the ultrasonography density of testicular parenchyma in pixels, the greater the sperm concentration. Our findings meet with the study made by Moxon et al. (2015), who measured the echogenicity of dogs’ testicles through ultrasonography, correlating the highest spermatic production with the ultrasonography heterogenicity, concluding that this complementary methodology is useful for evaluating fertility in specimens.

Evidence of the histological morphology evaluation, in birds of the TG it was shown a higher cellular proliferation at seminiferous tubules, which is consistent with the findings of Hänse et al. (2008), who evaluated through histological morphology the testicles of *M. undulatus* in reproductive season, finding that 78% of the evaluated testicles showed an active development of the germinal epithelium and evident cellular proliferation.

## Conclusion

The administration of GnRH-IS is a viable alternative to be used as part of the assisted reproduction techniques, to induce a production of gametes in male parakeet that may not be in their natural reproductive season or when reproductive activity is inhibited by captivity or imbued birds, with the purpose of increasing the parakeet reproduction on captivity for its preservation or sustainable economic use.

## List of abbreviations used

CG: Control Group
FSH: Follicle Stimulant Hormone
GnRH: Gonadotropin Release Hormone
GnRH-I: GnRH variant 1
GnRH-II: GnRH variant 2
GnRH-III: GnRH variant 3
GnRH-IS: GnRH-I analogs.
LH: Luteinizing Hormone.
M: Molar.
Pix: Pixels.
TG: Treated Group.
TSH: Thyroid Stimulant Hormone.
